# Neutrophil-to-Lymphocyte Ratio Predicts PSA Response and Prognosis in Prostate Cancer: A Systematic Review and Meta-Analysis

**DOI:** 10.1371/journal.pone.0158770

**Published:** 2016-07-01

**Authors:** Jian Cao, Xuan Zhu, Xiaokun Zhao, Xue-Feng Li, Ran Xu

**Affiliations:** 1 Department of Urology, The Second Xiangya Hospital, Central South University, Hunan Province, People’s Republic of China; 2 MRC Centre for Reproductive Health, Queen’s Medical Research Institute, Edinburgh, United Kingdom; University of North Carolina School of Medicine, UNITED STATES

## Abstract

An unprecedented advance has been seen in castration-resistant prostate cancer (CRPC) treatments in the past few years. With a number of novel agents were approved, there is a pressing need to develop improved prognostic biomarkers to facilitate the personalised selection and sequencing of these novel agents. Emerging evidence indicates that the neutrophil-to-lymphocyte ratio (NLR) is associated with poorer survival in patients with prostate cancer (PCa). However, the importance of the NLR for the prediction of the PSA response (PSARS) and biochemical recurrence (BCR) has been largely neglected. Here, we conducted a systematic review and meta-analysis to evaluate the prognostic value of the NLR for the PSARS, BCR, and survival in PCa. A systematic database search was performed using Embase, PubMed, the Cochrane Library, and the China National Knowledge Infrastructure (CNKI). A meta-analysis was performed by pooling hazard ratios (HRs), odds ratios (ORs) and the corresponding 95% confidence intervals (CIs). A total of 22 studies were included in the meta-analysis. Our results suggest that an elevated NLR predicts a lower PSARS rate (OR = 1.69, 95% CI: 1.40–1.98) and a higher possibility of BCR (HR = 1.12, 95% CI: 1.02–1.21). Additionally, we confirmed that an elevated NLR was a prognostic predictor of shorter overall survival (OS) in both metastatic castration-resistant PCa (mCRPC) (HR = 1.45, 95% CI: 1.32–1.59) and localized PCa (LPC) (HR = 1.12, 95% CI: 1.01–1.23) and that it predicted worse progression-free survival (PFS) in CRPC (HR = 1.42, 95% CI: 1.23–1.61) and poorer recurrence-free survival (RFS) (HR = 1.38, 95%CI: 1.01–1.75) in LPC. Our results suggest that an elevated NLR might be employed as a prognostic marker of biochemical changes and prognosis to facilitate risk stratification and decision making for individual treatment of PCa patients. The potential mechanisms underlying these associations and future research directions are also discussed.

## Introduction

Prostate cancer (PCa) is a major health concern for the male population; PCa is the second most common cancer and the fifth leading cause of cancer-associated death in men worldwide[1]. Although PCa-specific mortality in the US and the UK has declined markedly since the 1990s[2], slow increases and stable mortality have been observed in other regions[3]. A steady increase in the incidence rate was observed from 1975 to the early 1990s owing to the widespread application of transurethral resection and PSA testing for PCa screening[4,5]. This increase was followed by a stable incidence trend with an increased rate among patients younger than 70 years[6].

The PSA test was a landmark development in the early diagnosis of prostate cancer. Because PSA (a glycoprotein secreted by the prostate gland) is organ-derived and elevated PSA may also be caused by benign prostatic hypertrophy, prostatitis and recent manipulations of the prostate (massage, urethroscopy or biopsy), there is no precise threshold for a normal PSA value. However, higher PSA levels are thought to be indicative of a greater likelihood of PCa[7]. Some PSA-related testing parameters (e.g., PSA density, free/total PSA ratio, PSA doubling time, and prostate health index test) have been used to improve the accuracy of PCa prediction[7]. PSA is also important for monitoring biochemical recurrence (BCR) after radical therapies and managing metastatic disease[8].

With a number of new drugs have been approved for CRPC in the past few years, personalised drug selection and sequencing of agents has become more challenging. Therefore, prognostic biomarkers reflecting the response of this disease to these novel agents are needed to guide clinical management. The host inflammatory response plays a significant role in tumor progression[9]. Many parameters are utilized to determine the inflammatory response status of patients; the neutrophil-to-lymphocyte ratio (NLR) is the most commonly used because it is easily accessible. Many clinical studies have demonstrated that an increased NLR is correlated with poor prognosis in prostate cancer, and it also predicts PSA responses under different drug treatments. However, the results of these studies are inconsistent. A recent meta-analysis indicated that an elevated NLR was correlated with worse OS and PFS in mCRPC patients; however, this study did not consider biochemical changes in PCa[10]. To derive a more precise estimate of the prognostic significance of the NLR, in this study, we performed a systematic review of the most recently published studies. The importance of the NLR in predicting OS, PSARS and BCR in PCa were established using standard meta-analysis techniques.

## Materials and Methods

### Search strategy

Systematic document retrieval was performed using PubMed, Embase, the Cochrane Library and China National Knowledge Infrastructure (CNKI) for all relevant studies without restrictions. To reduce heterogeneity, we also retrieved relevant studies written in Chinese from CNKI. Literature addressing the prognostic value of the NLR was searched by combining the following terms: (“neutrophil lymphocyte ratio” OR “neutrophil to lymphocyte ratio” OR “neutrophil-to-lymphocyte ratio” OR “NLR”) AND (“prostate neoplasm” OR “prostate carcinoma” OR “prostatic cancer” OR “prostatic neoplasm” OR “prostatic carcinoma”).

The last search was updated on February 11, 2016. The bibliographies of the relevant articles were also explored to identify any studies missed by the electronic search strategies. The search was independently conducted by two authors, and any discrepancies were resolved through group discussion and establishing a consensus.

### Study selection and data extraction

After the removal of duplicates, two independent reviewers (including one urologist) screened the titles, abstracts, and full texts of all related articles using established criteria. The inclusion criteria were as follows: (a) confirmed diagnosis of PCa based on pathological examination; (b) evaluation of the prognostic value of the pretreatment NLR in PCa; (c) reported association between the NLR and PCa prognosis; and (d) reported HR, OR and 95% CI for prognosis or inclusion of data sufficient to estimate these statistics. The exclusion criteria were as follows: (a) reviews, case reports, letters, editorials, and animal and in vitro studies; (b) studies without sufficient data; (c) studies that did not include specific prognostic data concerning the NLR; and (d) articles written in languages other than Chinese or English.

The data were extracted using a reporting checklist proposed by the Meta-analysis of Observational Studies in Epidemiology (MOOSE) Group[11]. The NLR value was calculated as the absolute neutrophil count divided by the absolute lymphocyte count obtained from a pre-treatment blood sample in all included studies. To date, there has been no validated cut-off value for NLR. In this meta-analysis, seven eligible studies defined elevated NLR as NLR≥5 and five studies used NLR≥3 as elevated NLR and two studies used NLR≥2, another two studies used NLR≥2.6 and ≥3.5 respectively. The BCR was defined as a PSA level ≥0.2 ng/ml in two consecutive tests. PSARS was defined as a PSA decline of ≥50% from baseline that was maintained for longer than 3 weeks. The HR, OR and 95% CI were obtained directly from each study or from reconstruction according to the methods described by Tierney et al[12]. The logarithm of the HR (logHR) was assumed to have a normal distribution. If CIs were reported, SEs for the logHRs were calculated. Engauge Digitizer (4.1) was used to read the Kaplan-Meier curves and extract data for reconstruction if the survival data were only provided in the form of figures. The data extraction was independently performed by two investigators, and any discrepancies were resolved by group discussion and consensus.

### Statistical analysis

Standard meta-analysis methods[13] were applied to evaluate the overall effect of the NLR on the prognosis of PCa patients. Because the statistical methods (log-rank and Cox model) used in the eligible studies were inconsistent, the results were combined using the generic inverse variance method[13]. The consistency of the results (effect sizes) among studies was investigated using two heterogeneity tests: Cochran’s Q test and Higgins’s I-squared test. If heterogeneity was observed (Q test P value <0.10 and I^2^>50%), a meta-analysis was performed by applying the random-effects model[14]; otherwise, the fixed-effects model was applied.

Sensitivity to influential studies was evaluated by recalculating the pooled HRs after omitting each study from the meta-analysis consecutively (leave-one-out procedure). Subgroup analysis of the more homogeneous set of studies (i.e., those using the same stratification and study design features) was adopted as an additional sensitivity test.

Publication bias (i.e., negative trials are cited less frequently and therefore are more likely to be missed in the search for relevant studies) of the studies was analyzed using Begg’s funnel plot and Egger’s linear regression test. The “trim and fill” method was used to evaluate the influence of the missing publication on the overall effect[15]. Unless otherwise noted, no significant heterogeneity or publication bias was detected among the included studies. All statistical analyses were conducted with STATA version 12.0 software (STATA Corporation, College Station, TX, USA) and Revman version 5.3 software (Review Manager (RevMan) [Computer program]. Version [5.3]. Copenhagen: The Nordic Cochrane Centre, The Cochrane Collaboration, 2014). All statistical tests were two-sided, and the significance level was set at 5%.

## Results

### Literature searches and study characteristics

**Fig 1:**
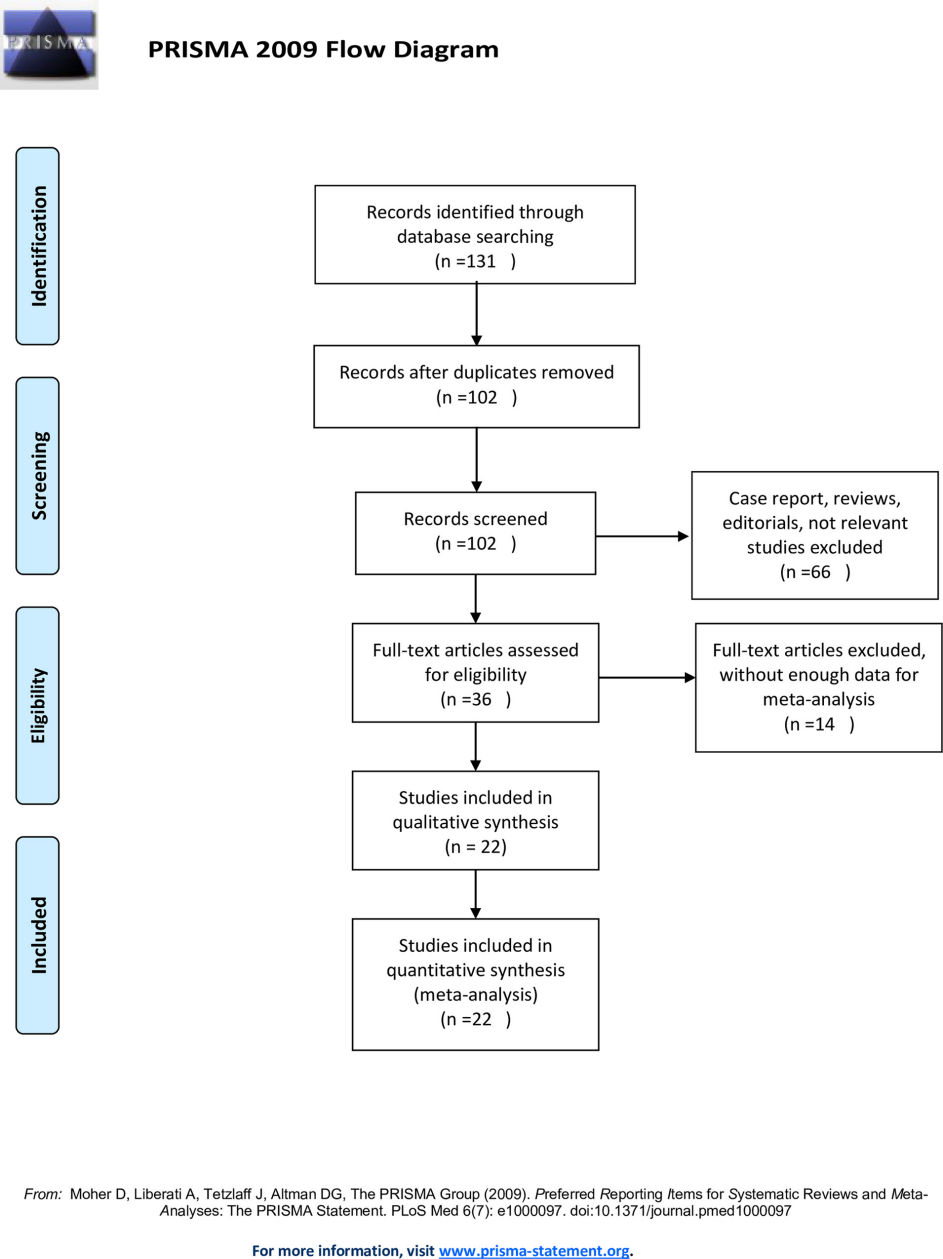
Flow chart outlining the study selection process.

The selection flow of the relevant articles is shown in Fig 1. The initial search algorithm retrieved 131 studies. After removal of duplicates, two investigators independently screened the titles and abstracts of the remaining studies according to the predetermined criteria. Thirty-six studies remained after excluding case reports, reviews, editorials and irrelevant studies. After the full texts and bibliographies were screened, another 14 studies were removed owing to the inclusion of insufficient survival data for the meta-analysis. Thus, 22 relevant studies[16–37] published from 2012 to 2015 were included in the meta-analysis. The characteristics of the included studies are summarized in S1 Table.

In the 22 selected studies concerning the association between the NLR and PCa prognosis, 16 cohorts informed the link between the NLR and OS, seven studies investigated the NLR and RFS, six cohorts reported a link between the NLR and PFS, six cohorts provided correlations between the NLR and PSARS, five studies reported an association between the NLR and BCR (S1 Table). A meta-analysis was employed for each end point.

### NLR and PSARS in mCRPC

Our meta-analysis detected a significant correlation between the NLR and PSARS. The pooled estimate from 6 cohorts indicated that patients with elevated pre-treatment NLRs tended to have 1.69-fold lower PSARSs after docetaxel- or cabazitaxel-based chemotherapy and androgen synthesis inhibitor (ASI) treatment (abiraterone or ketoconazole) compared to those patients with NLR below cut-off values(OR = 1.69, 95% CI: 1.40–1.98) (Table 1).

**Table 1:** Meta-analysis of NLRs based on different end points.

End Points	Cohorts	Patients	HR/OR (95% CI)	Heterogeneity^1^	Effects Model
**OS**	16	15298	HR = 1.40(1.25–1.55)	I^2^ = 60.0%, P = 0.001	Random
**PFS**	6	1629	HR = 1.42(1.23–1.61)	I^2^ = 2.4%, P = 0.401	Fixed
**BCR**	4	10171	HR = 1.12(1.02–1.21)	I^2^ = 11.7%, P = 0.339	Fixed
**PSARS**	6	3194	OR = 1.69(1.40–1.98)	I^2^ = 0.0%, P = 0.590	Fixed
**RFS**	7	11745	HR = 1.38(1.01–1.75)	I^2^ = 79.2%, P = 0.000	Random

^1^Heterogeneity was evaluated by Higgins I-squared test (I^2^) and Cochran’s Q test (p).

A p value of the Q test >0.10 and I^2^ <50% indicate homogeneity.

**Fig 2:**
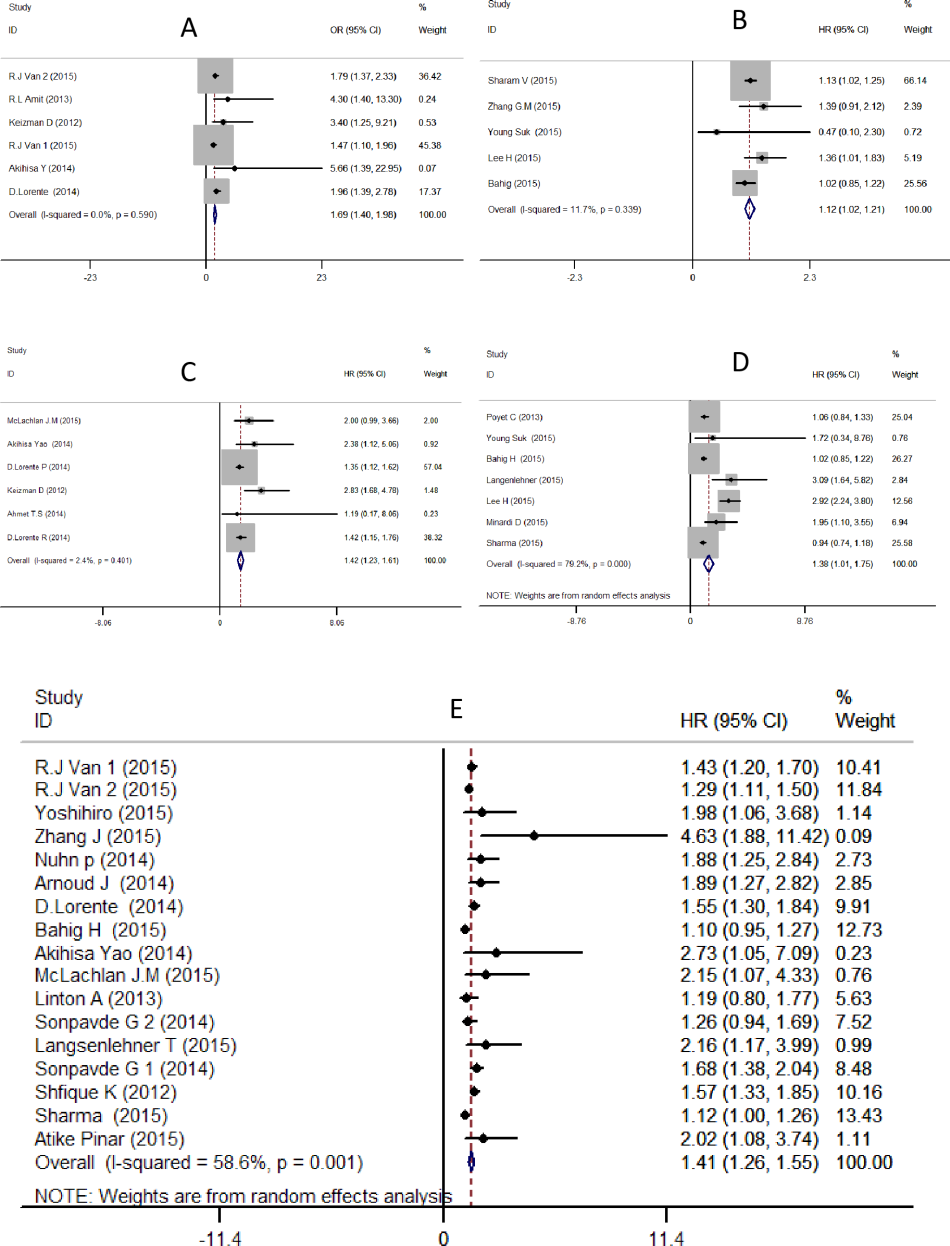
Forest plot and meta-analysis of studies evaluating the association between an elevated NLR and PSARS (A), BCR (B), PFS (C), RFS (D), OS (E).

Subgroup analysis by therapy was conducted. The patients in the two cohorts received ASI, whereas chemotherapy agents such as docetaxel or cabazitaxel were administered in the remaining cohorts. The association of elevated pre-treatment NLRs with lower PSARS was replicated in the cohorts that received chemotherapy (OR = 1.68, 95% CI: 1.39–1.97). However, an elevated NLR was not significantly associated with PSARS in patients who received ASI (OR = 3.68, 95% CI: 0.37–6.99) (Fig 2A)

### NLR and BCR in PCa after radical prostatectomy

Five studies investigated the association between an elevated NLR and BCR in PCa patients after radical prostatectomy. Two studies by Sharam V and Lee H revealed that patients with higher NLRs experienced BCR significantly sooner. The remaining two studies by Zhang G M and Young Suk identified a similar but not significant trend. However, a significant, albeit modest, effect was observed after integration into our meta-analysis, with a 1.12-fold higher BCR after radical prostatectomy in patients with an elevated preoperational NLR (HR = 1.12, 95% CI: 1.02–1.21) (Fig 2B) (Table 1).

### NLR and OS in PCa

Fourteen studies reported a correlation between pre-treatment NLR and OS in PCa patients. Two independent cohorts were included in the studies of R.J. Van et al and Sonpavde G et al; we labeled these cohorts R.J Van/TAX327, R.J Van/VENICE, Sonpavde G 1, and Sonpavde 2. Thus, a total of 16 cohorts were included in the OS analysis.

For the overall population, a random (I–V) analysis was selected due to heterogeneity (I^2^ = 60.0%, P = 0.001). The pooled HR of 1.40 (95% CI: 1.25–1.55) indicates that patients with elevated NLRs are expected to have shorter OS (Fig 2E) (Table 1).

In the subgroup analysis based on ethnicity, an elevated NLR appeared to be a stronger predictor of risk in Asian patients than Caucasian patients, with HRs of 2.25 (95% CI: 1.08–3.41) and 1.39 (95% CI: 1.24–1.53), respectively (Table 2). When the patients were stratified by status, an elevated NLR was significantly associated with OS in mCRPC and LPC patients, with HRs of 1.45 (95% CI: 1.32–1.59) and 1.12 (95% CI: 1.01–1.23), respectively (Table 2). Further subgroup analysis based on cut-off value (NLR≥5: HR = 1.40, 95% CI: 1.15–1.66 VS. NLR<5: HR = 1.41, 95% CI: 1.21–1.62) and sample size (n>300: HR = 1.39, 95% CI: 1.23–1.56, vs. n<300: HR = 1.43, 95% CI: 1.11–1.74) produced similar results (Table 2).

**Table 2:** Subgroup meta-analysis of the NLR and OS.

Subgroup	Factor	Cohort numbers	HR(95%CI)	Heterogeneity
**Ethnicity**	Caucasian	13	1.39(1.24–1.53)	I^2^ = 0.0%,P = 0.546
	Asian	3	2.25(1.08–3.41)	I^2^ = 64.3%,P = 0.001
**Patient Status**	mCRPC	12	1.45(1.32–1.59)	I^2^ = 16.2%,P = 0.286
	LPC	3	1.12(1.01–1.23)	I^2^ = 6.9%, P = 0.342
**Sample Size**	n>300	9	1.39(1.23–1.56)	I^2^ = 73.5%,P = 0.000
	n<300	7	1.43(1.11–1.74)	I^2^ = 10.7%,P = 0.348
**Cut-off value**	NLR≥5	7	1.40(1.15–1.66)	I^2^ = 68.3%,P = 0.004
	NLR<5	9	1.41(1.21–1.62)	I^2^ = 56.5%,P = 0.019

### NLR and PFS in CRPC

Six cohorts exhibited an association between an elevated NLR and PFS in CRPC. The pooled-effect estimates (HR = 1.42, 95% CI: 1.23–1.61) indicated a significant correlation between an elevated pre-treatment NLR and worse PFS in CRPC (Table 1) (Fig 2C).

### NLR and RFS in LPC after radical prostatectomy

Seven studies documented RFS data. Two of these studies reported that the NLR was a significant prognostic indicator, whereas the remaining studies did not observe a notable association between an elevated NLR and shorter RFS. However, our meta-analysis of these studies indicated a significant correlation between these factors, with a pooled HR of 1.38 (95% CI: 1.01–1.75, I^2^ = 79.2%, p = 0.00) (Table 1, Fig 2D) under the random-effects model.

#### Extensive statistical analysis

**Fig 3:**
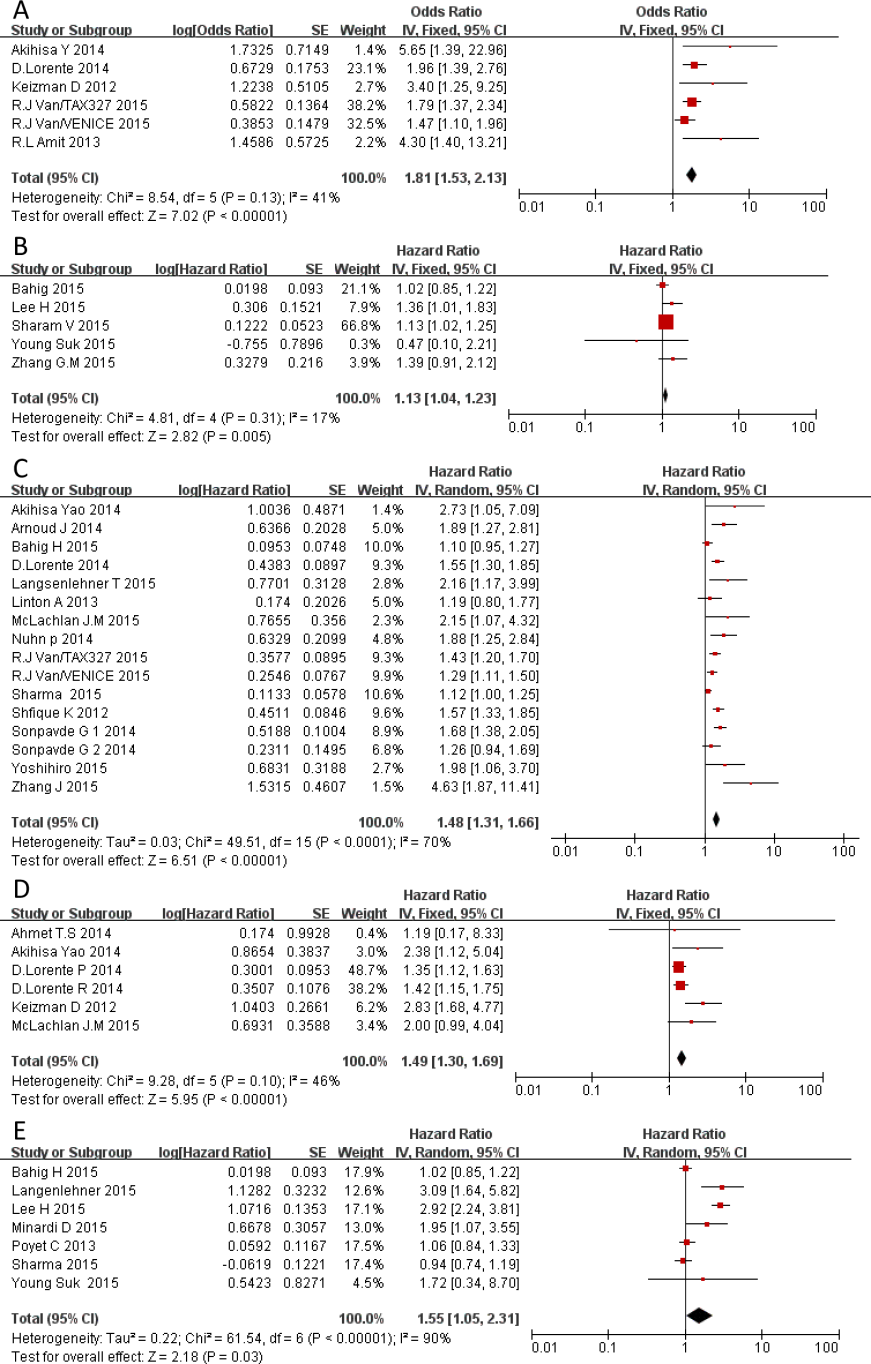
Forest plot and meta-analysis of studies evaluating the association between an elevated NLR and PSARS (A), BCR (B), PFS (C), RFS (D), OS (E).

To verify the above results obtained using Stata, another analysis was conducted using generic inverse variance method via Revman 5.3. Fig 3 showed similar results in PSARS, BCR, OS, RFS, and PFS analysis when generic inverse variance method was employed in Revman.

### Publication bias and heterogeneity evaluation

Begg’s funnel plot and Egger’s test were employed to assess the overt publication bias in each meta-analysis in this study. Publication biases were identified in the OS analysis by Egger’s test (p>|t| = 0.002) and in the PSARS analysis with Pr>|Z| = 0.024 by Begg’s funnel plot and p>|t| = 0.013 by Egger’s test. We applied “trim and fill” analysis to identify the source of the publication bias. It was estimated that there were five unpublished studies evaluating the role of the NLR in OS (S1A Fig) and three unpublished studies evaluating the role of NLR in PSARS (S1B Fig). The results of the filled meta-analysis that combined estimated unpublished studies correlated well with our primary pooled results with a pooled HR = 1.40 (95% CI: 1.25–1.57) for the NLR and OS and a pooled OR = 1.71 (95% CI: 1.46–2.01) for the NLR and PSARS. No obvious publication biases were detected in the other analyses.

Sensitivity analysis indicated that the majority of the heterogeneity in the OS analysis was contributed by the studies by Bahig H et al. and Sharma et al. (S2A Fig). The main source of heterogeneity in these two studies was the presentation of data from univariate analysis. After excluding these studies, the corresponding pooled HR of 1.46 (95% CI: 1.36–1.56) was not significantly altered and exhibited no obvious heterogeneity (I^2^ = 13.1%, p = 0.31), indicating the reliability of the results. Subgroup analysis performed as an additional sensitivity test also indicated the source of heterogeneity. Statistical methods (univariate analysis) were also the major contributor of heterogeneity in the RFS analysis (S2B Fig).

## Discussion

In the present study, we utilized 18,092 cases from 22 related studies to evaluate the prognostic role of the NLR in PCa. To the best of our knowledge, this is the first meta-analysis to investigate the relationship between an elevated NLR and PSARS, BCR. The results indicated that an elevated NLR was a mild risk factor for BCR in PCa patients after radical prostatectomy. Furthermore, we determined that an elevated NLR was a strong predictor of PSARS in mCRPC patients. An elevated NLR predicted worse OS, PFS, and RFS, in contrast to the results of a previous meta-analysis by Yin et al. These authors found that an elevated NLR was significantly associated with OS in mCRPC but not LPC. However, our analysis included more studies and thus more available evidence with less heterogeneity, and we demonstrated that an elevated NLR was significantly associated with OS not only in mCRPC but also in LPC. By contrast, a lower risk was observed in localized LPC.

A significant association between an elevated NLR and RFS in LPC was identified that was not reported in the previous study. The prognostic value of the NLR has been demonstrated in many malignancies[38–40]. An elevated NLR may be associated with both an increased neutrophil-dependent systemic inflammatory response and a lower lymphocyte-mediated antitumor immune response, reflecting a supportive tumor microenvironment[41,42]. Neutrophils are the predominant leukocyte subset in human peripheral blood and play an important role in tumor development by producing cytokines, proteases, and reactive oxygen species (ROS) and interacting with other immune cells[9]. Tumor-associated neutrophils can favor genetic instability via the release of ROS, promote tumor cell proliferation via elastase, sustain angiogenesis via the release of vascular endothelial growth factor (VEGF), enhance neoplastic cell invasiveness by secreting hepatocyte growth factor (HGF), oncostatin M (OSM)[43], and matrix metallopeptidase 9 (MMP-9), and suppress effective antitumor CD8^+^ T cell immunity via arginase expression[44]. By contrast, lymphocytes are critical components of antitumor immunity. CD8^+^ T lymphocytes, which recognize endogenous intracellular antigens presented by MHC class I molecules, are directly capable of killing tumor cells[45]. CD4^+^ T lymphocytes are central to immune system functions and play a vital role in tumor immunity. CD4^+^ T lymphocytes (also known as helper T lymphocytes) recognize antigens presented by MHC class II molecules, assist cytotoxic CD8^+^ T cells, and aid antibody production by B lymphocytes, thereby increasing the efficiency of tumor destruction[46]. Moreover, CD4^+^ T cells can directly or indirectly lyse tumor cells[46]. In addition to their antibody production capacity, B lymphocytes are involved in tumor surveillance by boosting T lymphocyte responses, serving as local antigen-presenting cells, and forming tertiary lymphoid structures in mutual cooperation with T cells and dendritic cells[47].

Recently, emerging studies have identified a heterogeneous population of myeloid-derived suppressor cells (MDSCs) that suppress tumour immunity responses and consequently significantly impede anti-cancer therapies. MDSCs are not a defined subset of myeloid cells, but rather a heterogeneous population of myeloid progenitor cells and immature myeloid cells[48]. In pathological conditions, such as cancer, MDSCs are expanded and activated by various factors produced by tumour cells and/or by stromal cells in the tumour microenvironment[48]. C. M. Diaz-Montero et al found that the percent and absolute number of MDSCs are significantly increased in the peripheral blood of patients with a range of solid tumours, including prostate cancer, than healthy volunteers[49]. More recently, two studies demonstrated that the CSF1R[50] and IL-6[51] pathways play critical roles in the migration of MDSCs to tumours microenvironments. MDSCs suppress tumour immunity mainly through its remarkable ability to suppress T-cell responses. Extensive studies have detailed how MDSCs mediated suppression of T-cell function through the release of enzymes (arginase 1 and iNOS), reactive oxygen species and peroxynitrite[48]. Furthermore, MDSCs also indirectly mediate T cell function by inducing T regulatory cells[48]. In addition to suppressing T cells, MDSCs also perturb tumour immunity in regulating macrophages and nature kill cells[52].

Hypothetically, the combined index of the elevated NLR likely reflects a favorable immune microenvironment for tumor development and metastasis. However, the exact mechanisms underlying the elevation in the NLR and the unfavorable outcomes are unknown. First, some T lymphocyte subsets have both pro- and antitumor properties. For example, CD4^+^ T lymphocytes can be further divided into TH_1_ cells, TH_2_ cells, TH_17_ cells and regulatory T cells. TH_17_ cells and regulatory T cells promote tumor progression. Because the NLR is a relative parameter, whether the increase in the NLR is due to a relative increase in neutrophils or decrease in lymphocytes in unclear. Importantly, changes in the lymphocyte subsets were not defined. Consequently, the NLR is an approximate index with heterogeneity that reflects the immune microenvironment for tumor development, which may explain the inconsistent results of the included studies. More specific NLR (e.g., NL_CD4TH2_R, NL_CD4TH17_R, and NL_B_R) indices should be explored to generate more precise predictions in future studies. Second, tumors are often infiltrated by various numbers of immune cells, which are also involved in cancer progression. However, the process of circulating immune cell recruitment to the tumor is not clear. Whether intra-tumor neutrophils are recruited from the bone marrow/blood pool of neutrophils or the spleen is unknown[53]. Moreover, neutrophils may influence the recruitment and differentiation of macrophages by releasing various cytokines[53].

Immune cells infiltrating prostate cancer tissues may be influenced by the tumor environment to facilitate PCa progression, which adds another level of complexity to the mechanisms of these cells in cancer development. Abundant immune cells have been detected in prostate tumor tissues by immunohistochemistry using different markers (CD3^+^, CD8^+^, CD20^+^, CD56^+^, CD68^+^ and Foxp3^+^)[54]. One study demonstrated that patients with very low and very high CD3^+^ T cell numbers had shorter BCR survival than patients with intermediate numbers of T cells; however, no significant association was identified when CD20 was used to mark B lymphocytes in the same patients[55]. Nora Ness et al. reported that the infiltration of high densities of CD8^+^ lymphocytes into prostate tumor epithelial areas was an independent risk factor for BCR [56]. Kiniwa et al. determined that regulatory T cells (CD8^+^ Foxp3^+^ or CD4^+^CD25^+^) present in prostate tumors mediate immunosuppression by suppressing naive T cell proliferation[57]. Furthermore, Vincenzo et al. revealed that cytotoxic T lymphocytes that infiltrated in the prostate tumor were immunosuppressed by substances secreted in the tumor microenvironment[58].

The potential of NLR to predict PSARS in response to drug treatments in mCRPC patients has been studied extensively, but less is known about the mechanisms. In our meta-analysis, a high NLR was associated with a low PSARS to chemotherapy but not ASI, thus suggesting that mCRPC patients with an elevated NLR may receive greater benefit from chemotherapy than ASI. Here, we hypothesize that different tumor immune environments after treatment with chemotherapy or ASI may be a contributing factor and subsequently affect the effectiveness of the treatments. Supporting this hypothesis, an increase in the relative densities of CD3^+^ and CD8^+^ T lymphocytes as well as CD68^+^ macrophages was observed under androgen depletion treatment compared to radical prostatectomy[54]. Additionally, NLR has been positively associated with PSA in men without prostatic disease[30] as well as with PCa[59]. McDonald et al. proposed that the NLR reflects the balance between innate (neutrophils) and adaptive (lymphocytes) immune responses; therefore, its association with higher serum PSA levels may indicate impairment in the adaptive host’s ability to control inflammation[30]. This finding may indicate that ASIs are more dependent on adaptive immune cells to fulfil their therapeutic effectiveness compared to chemotherapy.

Future studies should investigate the functions of specific subsets of neutrophils and lymphocytes due to the complex roles of immune cells in cancer development. This approach may facilitate the identification of new anticancer therapies or improve the effectiveness of existing treatments. The relationship between circulating immune cells and tumor-infiltrating immune cells is another vital problem. The mechanisms by which immune cells are recruited into tumor tissues to promote tumor progression should also be considered as therapeutic targets. The mechanisms underlying an elevated NLR and the response to anticancer treatment should also be explored. Such studies would facilitate the selection of patients who are more lost likely to benefit from drugs developed for mCRPC.

Our study has several limitations that should be carefully considered. First, studies lacking sufficient survival data for the meta-analysis were excluded. Second, the number of relevant studies was not sufficient to obtain a robust conclusion in some endpoint analyses. Another limitation of this study is the variety of NLR cut-off values employed in related studies. Further prospective studies are needed to confirm these conclusions.

## Conclusion

In conclusion, this meta-analysis provides evidence that an elevated NLR predicts lower PSARS after chemotherapy and poor survival outcomes in PCa. These two inexpensive and easily accessible indicators can help stratify high-risk patients and guide therapy choices. Future studies should seek to identify more specific NLRs for consistent and precise predictions. A greater understanding of the mechanisms underlying the role of the NLR in PCa progression will facilitate the development of new anticancer strategies.

## Supporting Information

S1 Fig Funnel plot adjusted with trim and fill method. A: Trim and Fill analysis of NLR and OS; B: Trim and Fill analysis of NLR and PSARS. Circles: included studies. Diamonds: presumed missing studies.
(TIF)

S2 Fig Heterogeneity resource evaluation. A: Sensitive analysis of NLR and OS; B: Sensitive analysis of NLR and RFS.
(TIF)

S1 File PRISMA 2009 checklist. (DOC)

S2 File Cover letter from Nature Publication Group language edition team for the first edition of this manuscript. (PDF)

S3 File Marked-up copy after edited by Nature Publication Group language edition team. (DOCX)

S1 Table Characteristics of all studies. (XLS)
