# Supplementary figures and images for: Neutrophil-to-Lymphocyte Ratio Predicts PSA Response and Prognosis in Prostate Cancer: A Systematic Review and Meta-Analysis
Source: PLoS One. 2016 Jul 1;11(7):e0158770. doi: 10.1371/journal.pone.0158770 (PMC4930176; doi:10.1371/journal.pone.0158770)

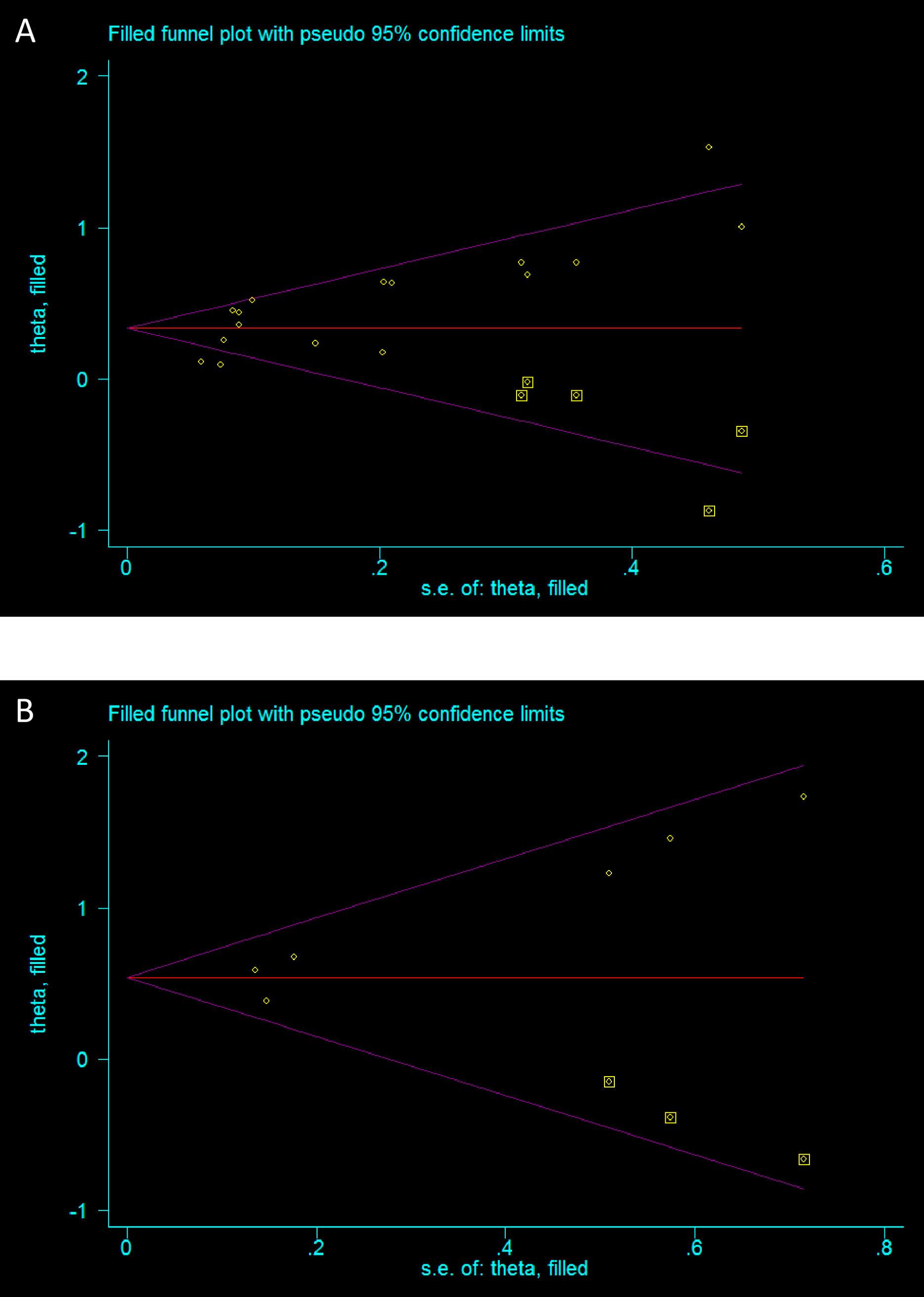

Supplement: S1 Fig — A: Trim and Fill analysis of NLR and OS; B: Trim and Fill analysis of NLR and PSARS. Circles: included studies. Diamonds: presumed missing studies. (TIF) [file pone.0158770.s001.tif]

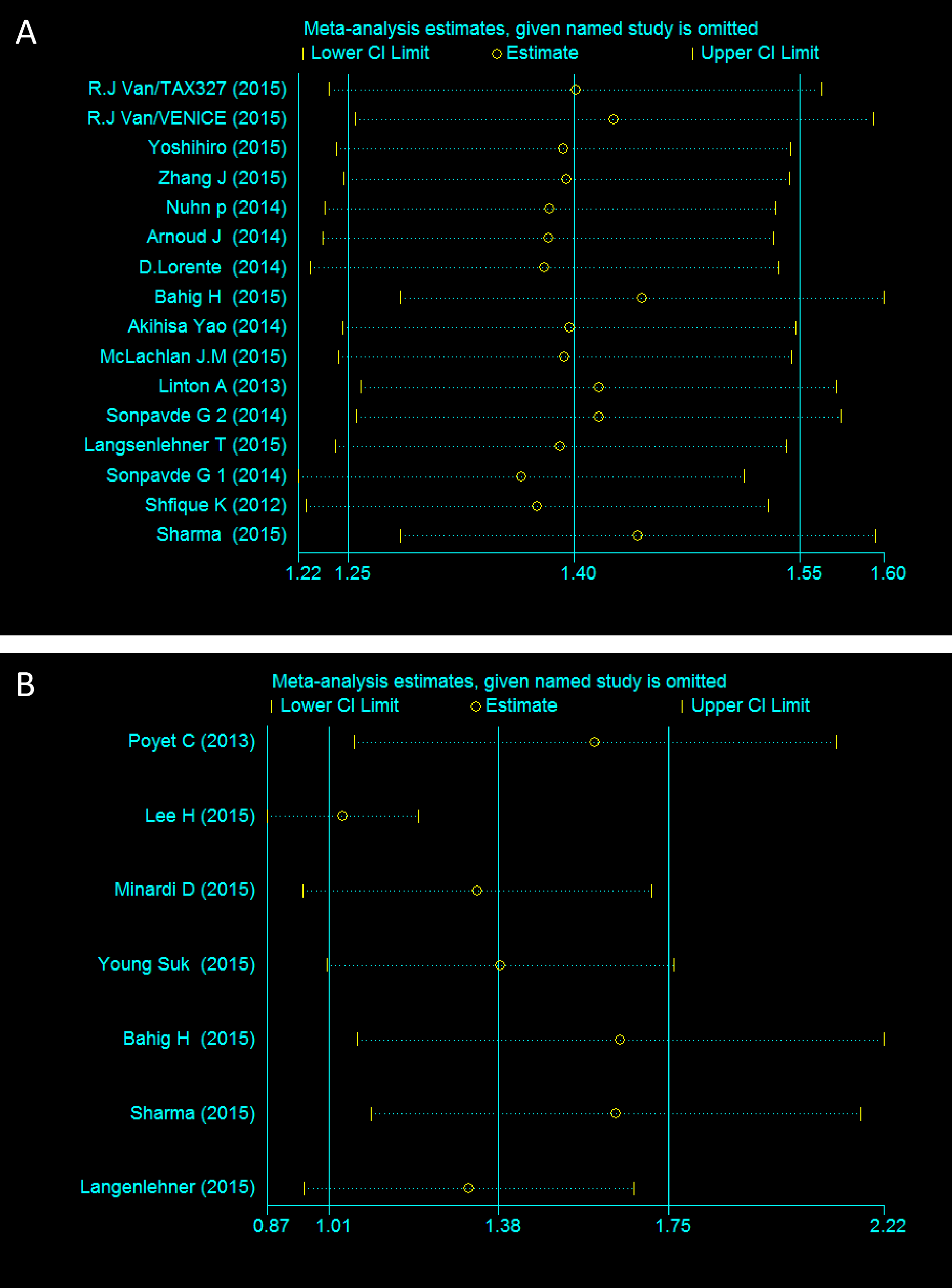

Supplement: S2 Fig — A: Sensitive analysis of NLR and OS; B: Sensitive analysis of NLR and RFS. (TIF) [file pone.0158770.s002.tif]
