# Supplementary material for: Neutrophil-to-Lymphocyte Ratio Predicts PSA Response and Prognosis in Prostate Cancer: A Systematic Review and Meta-Analysis
Source: PLoS One. 2016 Jul 1;11(7):e0158770. doi: 10.1371/journal.pone.0158770 (PMC4930176; doi:10.1371/journal.pone.0158770)
Supplement: S2 File — (PDF) [file pone.0158770.s004.pdf]

npg nature publishing group language editing
Premium science and medical editing

April 16, 2016

Dear Shi Zeng,

Thank you for choosing NPG Language Editing. The manuscript “Neutrophil-to-lymphocyte ratio predicts PSA response and prognosis in prostate cancer: A systematic review and meta-analysis” reports that an elevated neutrophil-to-lymphocyte ratio may be a prognostic marker of biochemical changes in prostate cancer with implications for treatment decisions. We have edited your manuscript by making changes to grammar and mechanics as well as improving style and flow. Below, I have used examples from the text to illustrate the types of revisions that were made.

Clarity: Language and sentence structure were revised to convey your intended meaning more clearly. For example, “A similar finding was replicated to cohorts received chemotherapy” was revised to “The association of elevated pre-treatment NLRs with lower PSARS was replicated in the cohorts that received chemotherapy”. The meaning of “a similar finding” in the original text was not clear because findings were not discussed in the sentence immediately preceding the revised sentence.

Formal language: Informal language was revised to more formal or specific language. For example, “showed” was revised to “exhibited” in the revision “Six cohorts exhibited an association between an elevated NLR and PFS in CRPC”. “Shown” was revised to “detected” in the revision “No obvious publication biases were detected in the other analyses”. “We are not sure if the raise of NLR comes from a relative increasing of neutrophils of a decrease of lymphocytes” was revised to “whether the increase in the NLR is due to a relative increase in neutrophils or decrease in lymphocytes in unclear”. Please note the revision of “not sure” to “unclear” and “raise” to “increase”.

“That” and “which”: In formal American writing, “that” is used restrictively to narrow a class or identify a particular item being discussed. Because “that” introduces a restrictive clause, such a clause is neither preceded nor followed by a comma. “Which” is generally used nonrestrictively—not to narrow a class or identify a particular item but rather to add something about the item already identified. When used nonrestrictively, the relative clause introduced by “which” should be enclosed in commas or, if at the end of a sentence, preceded by a comma. For example, “which” was revised to “that” in the revision “The results of the filled meta-analysis that combined estimated unpublished studies correlated well with our primary pooled results with a pooled HR=1.40 (95% CI: 1.25–1.57) for the NLR and OS and a pooled OR=1.71 (95% CI: 1.46–2.01) for the NLR and PSARS”. “That” is necessary to restrict the category of “filled meta-analysis” to only the meta-analysis that estimated unpublished studies.

Comments were left in several cases where further clarification would be helpful or confirmation of the meaning of the text was necessary. Please review these comments and all our changes carefully for more detailed feedback and suggestions, as well as to ensure that the final version of the manuscript is fully accurate.

Thank you again for using our editing services; we wish you the best of luck with your submission.

Best Regards,

Dawn S.
Senior Editor
NPG Language Editing

601 West Main Street, Suite 102 | Durham, NC 27701, United States | Tel: + | Fax: +1-919-287-2439 | languageediting.nature.com
