# Supplementary material for: Neutrophil-to-Lymphocyte Ratio Predicts PSA Response and Prognosis in Prostate Cancer: A Systematic Review and Meta-Analysis
Source: PLoS One. 2016 Jul 1;11(7):e0158770. doi: 10.1371/journal.pone.0158770 (PMC4930176; doi:10.1371/journal.pone.0158770)
Supplement: S3 File — (DOCX) [file pone.0158770.s005.docx]

**Neutrophil****-to-lymphocyte ratio predicts PSA response and prognosis in prostate cancer: A systematic review and meta-analysis**

**Jian Cao^1, 2 ¶^, Xuan Zhu^1 ¶^, Xue-Feng Li^2^, Xiaokun Zhao^1^ and Ran Xu^1 *^**

^1^Department of Urology, The Second Xiangya Hospital, Central South University, Hunan Province, People’s Republic of China;

^2^MRC Centre for Reproductive Health, Queen’s Medical Research Institute, Edinburgh EH16 4TJ, United Kingdom.

^*^Corresponding author

Email: ddf20@sina.com

**^¶^**These authors contributed equally to this work.

**Abstract**

Emerging evidence indicates that the neutrophil-to-lymphocyte ratio (NLR) is associated with poorer survival in patients with prostate cancer (PCa). However, the importance of the NLR for the prediction of the PSA response (PSARS) and biochemical recurrence (BCR) has been largely neglected. Here, we conducted a systematic review and meta-analysis to evaluate the prognostic value of the NLR for the PSARS, BCR, lymph node invasion (LNI), pathological stage (pT) and survival in PCa. A systematic database search was performed using Embase, PubMed, the Cochrane Library, and the China National Knowledge Infrastructure (CNKI). A meta-analysis was performed by pooling hazard ratios (HRs), odds ratios (ORs) and the corresponding 95% confidence intervals (CIs). A total of 22 studies were included in the meta-analysis. Our results suggest that an elevated NLR predicts a lower PSARS rate (OR=1.69, 95% CI: 1.40–1.98) and a higher possibility of BCR (HR=1.12, 95% CI: 1.02–1.21). Additionally, we confirmed that an elevated NLR was a prognostic predictor of shorter overall survival (OS) in both metastatic castration-resistant PCa (mCRPC) (HR=1.45, 95% CI: 1.32–1.59) and localized PCa (LPC) (HR=1.12, 95% CI: 1.01–1.23) and that it predicted worse progression-free survival (PFS) in CRPC (HR=1.42, 95% CI: 1.23–1.61) and poorer recurrence-free survival (RFS) (HR=1.38, 95%CI: 1.01–1.75) in LPC. Our results suggest that an elevated NLR might be employed as a prognostic marker of biochemical changes and prognosis to facilitate risk stratification and decision making for individual treatment of PCa patients. The potential mechanisms underlying these associations and future research directions are also discussed.

**Introduction**

Prostate cancer (PCa) is a major health concern for the male population; PCa is the second most common cancer and the fifth leading cause of cancer-associated death in men worldwide[1]. Although PCa-specific mortality in the US and the UK has declined markedly since the 1990s[2], slow increases and stable mortality have been observed in other regions[3]. A steady increase in the incidence rate was observed from 1975 to the early 1990s owing to the widespread application of transurethral resection and PSA testing for PCa screening[4,5]. This increase was followed by a stable incidence trend with an increased rate among patients younger than 70 years[6].

The PSA test was a landmark development in the early diagnosis of prostate cancer. Because PSA (a glycoprotein secreted by the prostate gland) is organ-derived and elevated PSA may also be caused by benign prostatic hypertrophy, prostatitis and recent manipulations of the prostate (massage, urethroscopy or biopsy), there is no precise threshold for a normal PSA value. However, higher PSA levels are thought to be indicative of a greater likelihood of PCa[7]. Some PSA-related testing parameters (e.g., PSA density, free/total PSA ratio, PSA doubling time, and prostate health index test) have been used to improve the accuracy of PCa prediction[7]. PSA is also important for monitoring biochemical recurrence (BCR) after radical therapies and managing metastatic disease[8].

The host inflammatory response plays a significant role in tumor progression[9]. Many parameters are utilized to determine the inflammatory response status of patients; the neutrophil-to-lymphocyte ratio (NLR) is the most commonly used because it is easily accessible. Many clinical studies, including those focused on PCa, have demonstrated that an increased NLR is correlated with poor prognosis in various cancer types[10–15]. However, the results of these studies are inconsistent. A recent meta-analysis indicated that an elevated NLR was correlated with worse OS and PFS in mCRPC patients; however, this study did not consider biochemical changes in PCa[16]. To derive a more precise estimate of the prognostic significance of the NLR, in this study, we performed a systematic review of the most recently published studies. The importance of the NLR in predicting OS, PSARS and BCR in PCa were established using standard meta-analysis techniques.

**Methods and materials**

**Search strategy**

Systematic document retrieval was performed using PubMed, Embase, the Cochrane Library and China National Knowledge Infrastructure (CNKI) for all relevant studies without restrictions. To reduce heterogeneity, we also retrieved relevant studies written in Chinese from CNKI. Literature addressing the prognostic value of the NLR was searched by combining the following terms: (“neutrophil lymphocyte ratio” OR “neutrophil to lymphocyte ratio” OR “neutrophil-to-lymphocyte ratio” OR “NLR”) AND (“prostate neoplasm” OR “prostate carcinoma” OR “prostatic cancer” OR “prostatic neoplasm” OR “prostatic carcinoma”).

The last search was updated on February 11, 2016. The bibliographies of the relevant articles were also explored to identify any studies missed by the electronic search strategies. The search was independently conducted by two authors, and any discrepancies were resolved through group discussion and establishing a consensus.

**Study selection and data extraction**

After the removal of duplicates, two independent reviewers (including one urologist) screened the titles, abstracts, and full texts of all related articles using established criteria. The inclusion criteria were as follows: (a) confirmed diagnosis of PCa based on pathological examination; (b) evaluation of the prognostic value of the pretreatment NLR in PCa; (c) reported association between the NLR and PCa prognosis; and (d) reported HR, OR and 95% CI for prognosis or inclusion of data sufficient to estimate these statistics. The exclusion criteria were as follows: (a) reviews, case reports, letters, editorials, and animal and in vitro studies; (b) studies without sufficient data; (c) studies that did not include specific prognostic data concerning the NLR; and (d) articles written in languages other than Chinese or English.

The data were extracted using a reporting checklist proposed by the Meta-analysis of Observational Studies in Epidemiology (MOOSE) Group[17]. The NLR value was calculated as the absolute neutrophil count divided by the absolute lymphocyte count obtained from a pre-treatment blood sample. The BCR was defined as a PSA level ≥0.2 ng/ml in two consecutive tests. PSARS was defined as a PSA decline of ≥50% from baseline that was maintained for longer than 3 weeks. The HR, OR and 95% CI were obtained directly from each study or from reconstruction according to the methods described by Tierney et al[18]. Engauge Digitizer (4.1) was used to read the Kaplan-Meier curves and extract data for reconstruction if the survival data were only provided in the form of figures. The data extraction was independently performed by two investigators, and any discrepancies were resolved by group discussion and consensus.

**Statistical analysis**

Standard meta-analysis methods[19] were applied to evaluate the overall effect of the NLR on the prognosis of PCa patients. Because the statistical methods (log-rank and Cox model) used in the eligible studies were inconsistent, the results were combined using the generic inverse variance method[19]. The consistency of the results (effect sizes) among studies was investigated using two heterogeneity tests: Cochran’s Q test and Higgins’s I-squared test. If heterogeneity was observed (Q test P value <0.10 and I^2^>50%), a meta-analysis was performed by applying the random-effects model[20]; otherwise, the fixed-effects model was applied.

Sensitivity to influential studies was evaluated by recalculating the pooled HRs after omitting each study from the meta-analysis consecutively (leave-one-out procedure). Subgroup analysis of the more homogeneous set of studies (i.e., those using the same stratification and study design features) was adopted as an additional sensitivity test.

Publication bias (i.e., negative trials are cited less frequently and therefore are more likely to be missed in the search for relevant studies) of the studies was analyzed using Begg’s funnel plot and Egger’s linear regression test. The “trim and fill” method was used to evaluate the influence of the missing publication on the overall effect[21]. Unless otherwise noted, no significant heterogeneity or publication bias was detected among the included studies. All statistical analyses were conducted with STATA version 12.0 software (STATA Corporation, College Station, TX, USA). All statistical tests were two-sided, and the significance level was set at 5%.

**Results**

**Literature searches and study characteristics**

The selection flow of the relevant articles is shown in Fig 1 (flow chart outlining the study selection process). The initial search algorithm retrieved 131 studies. After removal of duplicates, two investigators independently screened the titles and abstracts of the remaining studies according to the predetermined criteria. Thirty-six studies remained after excluding case reports, reviews, editorials and irrelevant studies. After the full texts and bibliographies were screened, another 14 studies were removed owing to the inclusion of insufficient survival data for the meta-analysis. Thus, 22 relevant studies[11,15,22–41] published from 2012 to 2015 were included in the meta-analysis. The characteristics of the included studies are summarized in Table S1.

In the 22 selected studies concerning the association between the NLR and PCa prognosis, 16 cohorts informed the link between the NLR and OS, seven studies investigated the NLR and RFS, six cohorts reported a link between the NLR and PFS, six cohorts provided correlations between the NLR and PSARS, five studies reported an association between the NLR and BCR, three studies investigated the NLR and pT1-2 vs. pT3-4, and two studies analyzed the LNI (Table S1). A meta-analysis was employed for each end point.

**NLR and PSARS in mCRPC**

Our meta-analysis detected a significant correlation between the NLR and PSARS. The pooled estimate from 6 cohorts indicated that patients with elevated pre-treatment NLRs tended to have 1.69-fold lower PSARSs after docetaxel- or cabazitaxel-based chemotherapy compared to androgen synthesis inhibitor (ASI) treatment (abiraterone or ketoconazole) (OR=1.69, 95% CI: 1.40–1.98) (Table 1).

**Table 1 Meta-analysis of NLRs based on different end points**

| **End Points** | **Cohorts** | **Patients** | **HR/OR (95% CI)** | **Heterogeneity^1^** | **Effects Model** |
| --- | --- | --- | --- | --- | --- |
| **OS**  **PFS**  **BCR**  **PSARS**  **RFS**  **pT**  **LNI** | 16  6  4  6  7  3  2 | 15298  1629  10171  3194  11745  1821  1604 | HR=1.40(1.25–1.55)  HR=1.42(1.23–1.61)  HR=1.12(1.02–1.21)  OR=1.69(1.40–1.98)  HR=1.38(1.01–1.75)  OR= 1.51(0.89–2.12)  OR=1.36(0.78–1.95) | I^2^=60.0%, P=0.001  I^2^=2.4%, P=0.401  I^2^=11.7%, P=0.339  I^2^=0.0%, P=0.590  I^2^=79.2%, P=0.000  I^2^=60.8%, P=0.078  I^2^=49.3%, P=0.160 | Random  Fixed  Fixed  Fixed  Random  Random  Fixed |

^1^Heterogeneity was evaluated by Higgins I-squared test (I^2^) and Cochran’s Q test (p). A p value of the Q test >0.10 and I^2^ <50% indicate homogeneity.

Subgroup analysis by therapy was conducted. The patients in the two cohorts received ASI, whereas chemotherapy agents such as docetaxel or cabazitaxel were administered in the remaining cohorts. The association of elevated pre-treatment NLRs with lower PSARS was replicated in the cohorts that received chemotherapy (OR=1.68, 95% CI: 1.39–1.97). However, an elevated NLR was not significantly associated with PSARS in patients who received ASI (OR=3.68, 95% CI: 0.37–6.99) (Fig 2: Forest plot and meta-analysis of studies evaluating the association between an elevated NLR and PSARS).

**NLR and BCR in PCa after radical prostatectomy**

Five studies investigated the association between an elevated NLR and BCR in PCa patients after radical prostatectomy. Two studies by Sharam V and Lee H revealed that patients with higher NLRs experienced BCR significantly sooner. The remaining two studies by Zhang G M and Young Suk identified a similar but not significant trend. However, a significant, albeit modest, effect was observed after integration into our meta-analysis, with a 1.12-fold higher BCR after radical prostatectomy in patients with an elevated preoperational NLR (HR=1.12, 95% CI: 1.02–1.21) (Fig 3: Forest plot and meta-analysis of studies evaluating the association between an elevated NLR and BCR) (Table 1).

**NLR and OS in PCa**

Fourteen studies reported a correlation between pre-treatment NLR and OS in PCa patients. Two independent cohorts were included in the studies of R.J. Van (2015) and Sonpavde G (2014); we labeled these cohorts R.J Van/TAX327, R.J Van/VENICE, Sonpavde G 1, and Sonpavde 2. Thus, a total of 16 cohorts were included in the OS analysis.

For the overall population, a random (I–V) analysis was selected due to heterogeneity (I^2^ =60.0%, P=0.001). The pooled HR of 1.40 (95% CI: 1.25–1.55) indicates that patients with elevated NLRs are expected to have shorter OS (Fig 4: Forest plot and meta-analysis of studies evaluating the association between an elevated NLR and OS) (Table 1).

In the subgroup analysis based on ethnicity, an elevated NLR appeared to be a stronger predictor of risk in Asian patients than Caucasian patients, with HRs of 2.25 (95% CI: 1.08–3.41) and 1.39 (95% CI: 1.24–1.53), respectively (Table 2). When the patients were stratified by status, an elevated NLR was significantly associated with OS in mCRPC and LPC patients, with HRs of 1.45 (95% CI: 1.32–1.59) and 1.12 (95% CI: 1.01–1.23), respectively (Table 2). Further subgroup analysis based on cut-off value (NLR≥5: HR=1.40, 95% CI: 1.15–1.66 VS. NLR<5: HR=1.41, 95% CI: 1.21–1.62) and sample size (n>300: HR=1.39, 95% CI: 1.23–1.56, vs. n<300: HR=1.43, 95% CI: 1.11–1.74) produced similar results (Table 2).

**Table 2 Subgroup meta-analysis of the NLR and OS**

| **Subgroup Factor Cohort numbers HR (95% CI) Heterogeneity Effects Model** |
| --- |
| **Ethnicity**  Caucasian 13 1.39(1.24–1.53) I^2^=0.0%, P=0.546 Random  Asian 3 2.25(1.08–3.41) I^2^=64.3%, P=0.001 Random  **Patient Status** mCRPC 12 1.45(1.32–1.59) I^2^=16.2%, P=0.286 Random  LPC 3 1.12(1.01–1.23) I^2^=6.9%, P=0.342 Random  **Sample Size** n>300 9 1.39(1.23–1.56) I^2^=73.5%, P=0.000 Random  n<300 7 1.43(1.11–1.74) I^2^=10.7%, P=0.348 Random  **Cut-off value** NLR≥5 7 1.40(1.15–1.66) I^2^=68.3%, P=0.004 Random  NLR<5 9 1.41(1.21–1.62) I^2^=56.5%, P=0.019 Random |

**NLR and PFS in CRPC**

Six cohorts exhibited an association between an elevated NLR and PFS in CRPC. The pooled-effect estimates (HR=1.42, 95% CI: 1.23–1.61) indicated a significant correlation between an elevated pre-treatment NLR and worse PFS in CRPC (Table 1).

**NLR and RFS in LPC after radical prostatectomy**

Seven studies documented RFS data. Two of these studies reported that the NLR was a significant prognostic indicator, whereas the remaining studies did not observe a notable association between an elevated NLR and shorter RFS. However, our meta-analysis of these studies indicated a significant correlation between these factors, with a pooled HR of 1.38 (95% CI: 1.01–1.75, I^2^=79.2%, p=0.00) (Table 1) under the random-effects model.

**NLR and** **pT in PCa after radical prostatectomy**

The association between a high NLR and pT progression was only investigated in three studies. Two of these studies (Zhang G M et al. and Lee H et al.) identified the NLR as a significant prognostic indicator of pT progression. By contrast, the study by Young Suk et al. detected a reverse correlation between these factors. The pooled OR of 1.51 (95% CI: 0.89–2.12, I^2^=60.8%, p=0.078) (Table 1) from the random-effects model indicated that an elevated NLR was not significantly associated with a higher pT.

**NLR and LNI in LPC after radical prostatectomy**

The two studies that reported an association between an elevated NLR and LNI reported contradictory results. The study by Zhang G.M et al. indicated that patients with higher NLRs had an increased risk of LNI. However, Lee H et al. observed a reverse correlation without a significant difference. The pooled OR value of 1.36 (95% CI: 0.76–1.95, I^2^ =49.3%, p=0.160) (Table 1) obtained after integrating these two studies together in the meta-analysis revealed a trend toward an elevated NLR as a risk factor for LNI that did not reach significance.

**Publication bias and heterogeneity evaluation**

Begg’s funnel plot and Egger’s test were employed to assess the overt publication bias in each meta-analysis in this study. Publication biases were identified in the OS analysis by Egger’s test (p>|t|=0.002) and in the PSARS analysis with Pr>|Z|=0.024 by Begg’s funnel plot and p>|t|=0.013 by Egger’s test. We applied “trim and fill” analysis to identify the source of the publication bias. It was estimated that there were five unpublished studies evaluating the role of the NLR in OS (Fig S1) and three unpublished studies evaluating the role of NLR in PSARS (Fig S2). The results of the filled meta-analysis that combined estimated unpublished studies correlated well with our primary pooled results with a pooled HR=1.40 (95% CI: 1.25–1.57) for the NLR and OS and a pooled OR=1.71 (95% CI: 1.46–2.01) for the NLR and PSARS. No obvious publication biases were detected in the other analyses.

Sensitivity analysis indicated that the majority of the heterogeneity in the OS analysis was contributed by the studies by Bahig H et al. and Sharma et al. (Fig S3). The main source of heterogeneity in these two studies was the presentation of data from univariate analysis. After excluding these studies, the corresponding pooled HR of 1.46 (95% CI: 1.36–1.56) was not significantly altered and exhibited no obvious heterogeneity (I^2^=13.1%, p=0.31), indicating the reliability of the results. Subgroup analysis performed as an additional sensitivity test also indicated the source of heterogeneity (Figs S4-7). Statistical methods (univariate analysis) were also the major contributor of heterogeneity in the RFS analysis (Fig S8).

**Discussion**

In the present study, we utilized 18,092 cases from 22 related studies to evaluate the prognostic role of the NLR in PCa. To the best of our knowledge, this is the first meta-analysis to investigate the relationship between an elevated NLR and PSARS, BCR, pT and LNI. The results indicated that an elevated NLR was a mild risk factor for BCR in PCa patients after radical prostatectomy but was not significantly associated with pT and LNI. Furthermore, we determined that an elevated NLR was a strong predictor of PSARS in mCRPC patients. An elevated NLR predicted worse OS, PFS, and RFS, in contrast to the results of a previous meta-analysis by Yin et al. These authors found that an elevated NLR was significantly associated with OS in mCRPC but not LPC. However, our analysis included more studies and thus more available evidence with less heterogeneity, and we demonstrated that an elevated NLR was significantly associated with OS not only in mCRPC but also in LPC. By contrast, a lower risk was observed in localized LPC. A significant association between an elevated NLR and RFS in LPC was identified that was not reported in the previous study.

The prognostic value of the NLR has been demonstrated in many malignancies[42–44]. An elevated NLR may be associated with both an increased neutrophil-dependent systemic inflammatory response and a lower lymphocyte-mediated antitumor immune response, reflecting a supportive tumor microenvironment[45,46]. Neutrophils are the predominant leukocyte subset in human peripheral blood and play an important role in tumor development by producing cytokines, proteases, and reactive oxygen species (ROS) and interacting with other immune cells[9]. Tumor-associated neutrophils can favor genetic instability via the release of ROS, promote tumor cell proliferation via elastase, sustain angiogenesis via the release of vascular endothelial growth factor (VEGF), enhance neoplastic cell invasiveness by secreting hepatocyte growth factor (HGF), oncostatin M (OSM)[47], and matrix metallopeptidase 9 (MMP-9), and suppress effective antitumor CD8^+^ T cell immunity via arginase expression[48]. By contrast, lymphocytes are critical components of antitumor immunity. CD8 T lymphocytes, which recognize endogenous intracellular antigens presented by MHC class I molecules, are directly capable of killing tumor cells[49]. CD4^+^ T lymphocytes are central to immune system functions and play a vital role in tumor immunity. CD4^+^ T lymphocytes (also known as helper T lymphocytes) recognize antigens presented by MHC class II molecules, assist cytotoxic CD8^+^ T cells, and aid antibody production by B lymphocytes, thereby increasing the efficiency of tumor destruction[50]. Moreover, CD4^+^ T cells can directly or indirectly lyse tumor cells[50]. In addition to their antibody production capacity, B lymphocytes are involved in tumor surveillance by boosting T lymphocyte responses, serving as local antigen-presenting cells, and forming tertiary lymphoid structures in mutual cooperation with T cells and dendritic cells[51].

Hypothetically, the combined index of the elevated NLR likely reflects a favorable immune microenvironment for tumor development and metastasis. However, the exact mechanisms underlying the elevation in the NLR and the unfavorable outcomes are unknown. First, some T lymphocyte subsets have both pro- and antitumor properties. For example, CD4^+^ T lymphocytes can be further divided into TH_1_ cells, TH_2_ cells, TH_17_ cells and regulatory T cells. TH_17_ cells and regulatory T cells promote tumor progression. Because the NLR is a relative parameter, whether the increase in the NLR is due to a relative increase in neutrophils or decrease in lymphocytes in unclear. Importantly, changes in the lymphocyte subsets were not defined. Consequently, the NLR is an approximate index with heterogeneity that reflects the immune microenvironment for tumor development, which may explain the inconsistent results of the included studies. More specific NLR (e.g., NL_CD4TH2_R, NL_CD4TH17_R, and NL_B_R) indices should be explored to generate more precise predictions in future studies. Second, tumors are often infiltrated by various numbers of immune cells, which are also involved in cancer progression. However, the process of circulating immune cell recruitment to the tumor is not clear. Whether intra-tumor neutrophils are recruited from the bone marrow/blood pool of neutrophils or the spleen is unknown[52]. Moreover, neutrophils may influence the recruitment and differentiation of macrophages by releasing various cytokines[52].

Immune cells infiltrating prostate cancer tissues may be educated by the tumor environment to facilitate PCa progression, which adds another level of complexity to the mechanisms of these cells in cancer development. Abundant immune cells have been detected in prostate tumor tissues by immunohistochemistry using different markers (CD3^+^, CD8^+^, CD20^+^, CD56^+^, CD68^+^ and Foxp3^+^)[53]. One study demonstrated that patients with very low and very high CD3^+^ T cell numbers had shorter BCR survival than patients with intermediate numbers of T cells; however, no significant association was identified when CD20 was used to mark B lymphocytes in the same patients[54]. Nora Ness et al. reported that the infiltration of high densities of CD8^+^ lymphocytes into prostate tumor epithelial areas was an independent risk factor for BCR [55]. Kiniwa et al. determined that regulatory T cells (CD8^+^ Foxp3^+^ or CD4^+^CD25^+^) present in prostate tumors mediate immunosuppression by suppressing naive T cell proliferation[56]. Furthermore, Vincenzo et al. revealed that cytotoxic T lymphocytes that infiltrated in the prostate tumor were immunosuppressed by substances secreted in the tumor microenvironment[57].

The potential of NLR to predict PSARS in response to drug treatments in mCRPC patients has been studied extensively, but less is known about the mechanisms. In our meta-analysis, a high NLR was associated with a low PSARS to chemotherapy but not ASI, thus suggesting that mCRPC patients with an elevated NLR may receive greater benefit from chemotherapy than ASI. Here, we hypothesize that different tumor immune environments after treatment with chemotherapy or ASI may be a contributing factor and subsequently affect the effectiveness of the treatments. Supporting this hypothesis, an increase in the relative densities of CD3^+^ and CD8^+^ T lymphocytes as well as CD68^+^ macrophages was observed under androgen depletion treatment compared to radical prostatectomy[53]. Additionally, NLR has been positively associated with PSA in men without prostatic disease[34] as well as with PCa[58]. McDonald et al. proposed that the NLR reflects the balance between innate (neutrophils) and adaptive (lymphocytes) immune responses; therefore, its association with higher serum PSA levels may indicate impairment in the adaptive host’s ability to control inflammation[34]. This finding may indicate that ASIs are more dependent on adaptive immune cells to fulfil their therapeutic effectiveness compared to chemotherapy.

Future studies should investigate the functions of specific subsets of neutrophils and lymphocytes due to the complex roles of immune cells in cancer development. This approach may facilitate the identification of new anticancer therapies or improve the effectiveness of existing treatments. The relationship between circulating immune cells and tumor-infiltrating immune cells is another vital problem. The mechanisms by which immune cells are recruited into tumor tissues to promote tumor progression should also be considered as therapeutic targets. The mechanisms underlying an elevated NLR and the response to anticancer treatment should also be explored. Such studies would facilitate the selection of patients who are more lost likely to benefit from drugs developed for mCRPC.

Our study has several limitations that should be carefully considered. First, studies lacking sufficient survival data for the meta-analysis were excluded. Second, the number of relevant studies was not sufficient to obtain a robust conclusion in some endpoint analyses. For instance, the results of the NLR and pT (n=3) and the LNI (n=2) evaluations suggested that an elevated NLR is a possible risk factor for pT and LNI, but the differences were not significant. Another limitation of this study is the variety of NLR cut-off values employed in related studies. Further prospective studies are needed to confirm these conclusions.

**Conclusion**

In conclusion, this meta-analysis provides evidence that an elevated NLR predicts lower PSARS after chemotherapy and poor survival outcomes in PCa. These two inexpensive and easily accessible indicators can help stratify high-risk patients and guide therapy choices. Future studies should seek to identify more specific NLRs for consistent and precise predictions. A greater understanding of the mechanisms underlying the role of the NLR in PCa progression will facilitate the development of new anticancer strategies.

**Acknowledgments**

We acknowledge all patients included in this study.

**Funding**

J Cao is sponsored by the China Scholarship Council (201506370137) as a visiting researcher at the University of Edinburgh.
